# Supplementary material for: Detecting long-range interactions between migrating cells
Source: Sci Rep. 2021 Jul 22;11:15031. doi: 10.1038/s41598-021-94458-0 (PMC8298713; doi:10.1038/s41598-021-94458-0)
Supplement: Supplementary file 1 — Supplementary Information 1. [file 41598_2021_94458_MOESM1_ESM.pdf]

Supplemental Information

Detecting long-range interactions between migrating cells

Claus Metzner*, Franziska Hörsch*, Christoph Mark*,
Tina Czerwinski*, Alexander Winterl*,
Caroline Voskens+, and Ben Fabry*

*Biophysics, Friedrich-Alexander University Erlangen-Nürnberg
+Dermatology, University Hospital Erlangen

Correspondence to *claus.metzner@gmail.com*

Data measured with NK and K562 cells in flat collagen gels

In addition to the data presented in the main part of the paper, we have analyzed 9 additional data sets DS0-DS8 (see table 1). The two human donors of the NK cells used in these 9 data sets were different from those in the main part of the paper. The experiments were this time performed in thin, quasi-two-dimensional matrices, and also the cell tracking was performed in a different way:

In data sets DS0-DS8, the NK cells were again in vitro activated and expanded. A number of $5 \cdot 10^6$ NK immune cells and $3 \cdot 10^6$ K562 tumor cells are mixed with ice-cold 500 μl acid-dissolved collagen solution (1.2mg/ml) and pipetted into a tissue-culture-treated 35 mm dish (Fig. 1(a); for a detailed protocol, see [**?**]). The polymerization of the collagen solution is initiated by placing the dish for 30 min in a cell culture incubator at 37°C, 5% CO_2. Due to surface tension, the thickness of the polymerized collagen gel decreases towards the center of the dish with a height of ≈ 30 μm (Fig. 1(b)). Time-lapse imaging can thus be realized in bright-field mode without scanning in z-direction, while the cells still showed the same characteristic migration behavior as in a thick collagen gel. We recorded 9 independent data sets, each including between 333 and 1547 images, with a time interval of 45 seconds between two subsequent frames. The images had 1344 x 1024 pixels with a linear size of 0.645 μm.

In data sets DS0-DS8, cells are automatically segmented using local differences of image entropy. The classification in immune and tumor cells is based on differences in speed, size, and brightness (Fig. 1(c)). Finally, the temporal trajectory of each cell is determined using the overlap of the cell area between successive time frames.

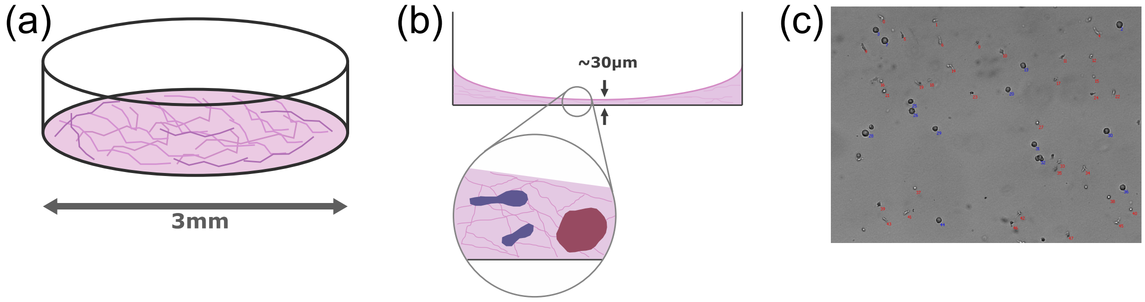

Figure 1: (a,b) Experimental setup for flat gels. (c) Example frame with NK cells in red and K562 cells in blue.

DS	N_{fra}	T_{rec} (h)	N_{tri}	$N_{imm}^{(0)}$	$N_{tum}^{(0)}$	$\overline{v} = \frac{\overline{\sigma}}{\Delta t}$ $(\frac{\mu m}{min})$	$\overline{\kappa}$
0	334	4.18	12075	34	14	5.96	2.59
1	414	5.18	6910	23	27	4.87	2.71
2	641	8.01	11166	22	17	6.75	2.37
3	1548	19.35	58736	34	14	5.07	2.57
4	633	7.91	29101	47	18	4.12	1.73
5	640	8.00	24272	26	26	6.39	3.28
6	640	8.00	10561	16	20	5.29	2.72
7	640	8.00	12590	19	23	6.20	2.67
8	640	8.00	8095	13	23	4.92	2.59

Table 1: Essential properties of the nine data sets $DS = 0 \ldots 8$. Here, N_{fra} is the number of video frames, T_{rec} the total recording time in hours, N_{tri} the number of valid triplets that could be used for the p-value evaluation, $N_{imm}^{(0)}$ the initial number of immune cells, $N_{tum}^{(0)}$ the initial number of tumor cells, $\overline{v}$ the average speed of immune cells in μm/min, and $\overline{\kappa}$ is the average persistence parameter of immune cells.

p-value distributions of NK cells with wildtype K562 cells (data sets DS0-DS8)

We do not find evidence for interactions between NK cells and wild-type K562 cells in any of the data sets DS0-DS8 (Fig. 2).

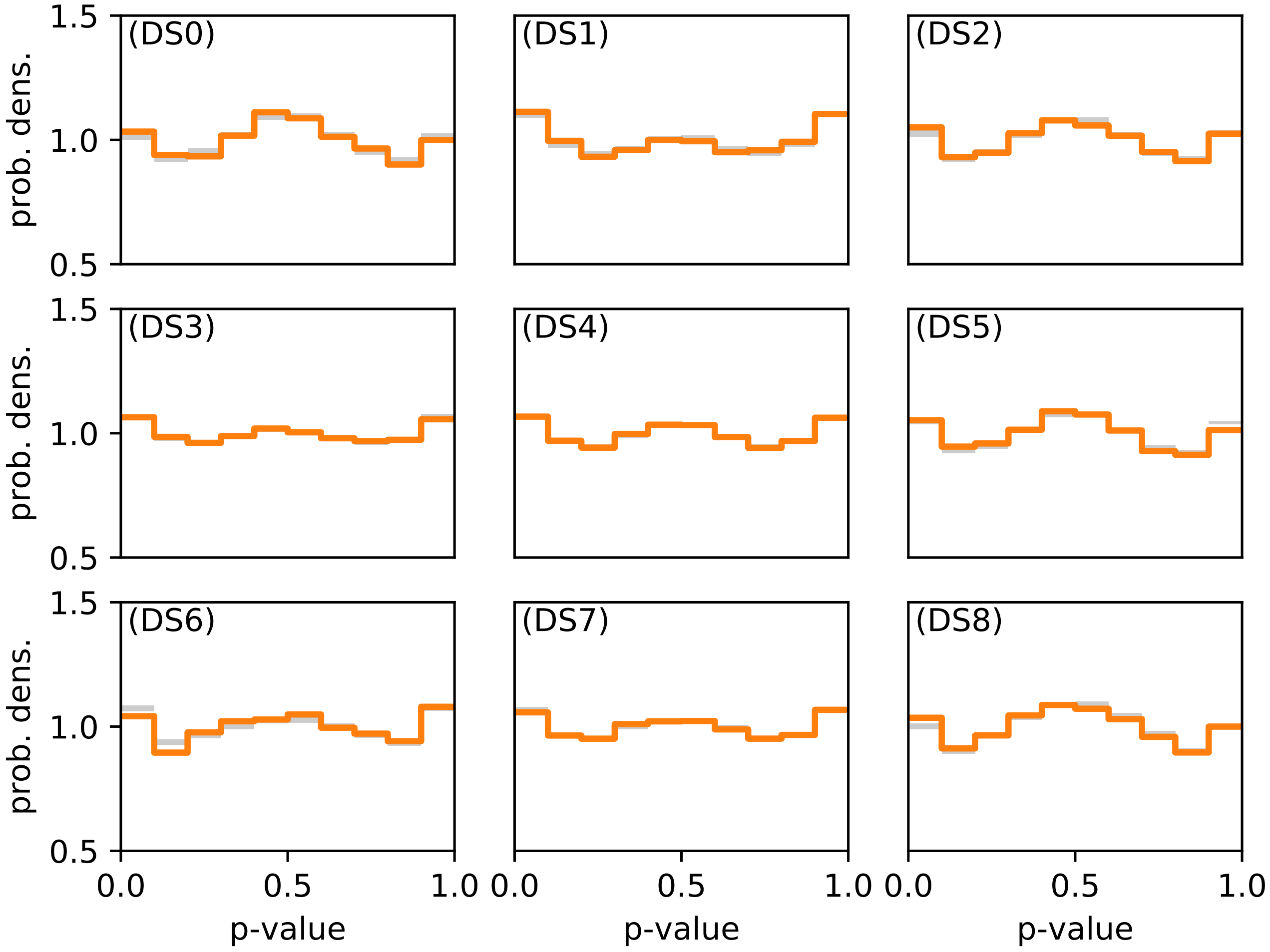

Figure 2: p-value distributions for data sets DS0-DS8, using a recording time interval of $\Delta t = 0.75$min and a maximum interaction radius of $r_{max} = 700\mu$m.

Effect of recording interval and max. interaction distance (data set DS0)

We do not find evidence for interactions between NK cells and wild-type K562 cells, independent of the recording interval and the maximum interaction distance (Fig. 3).

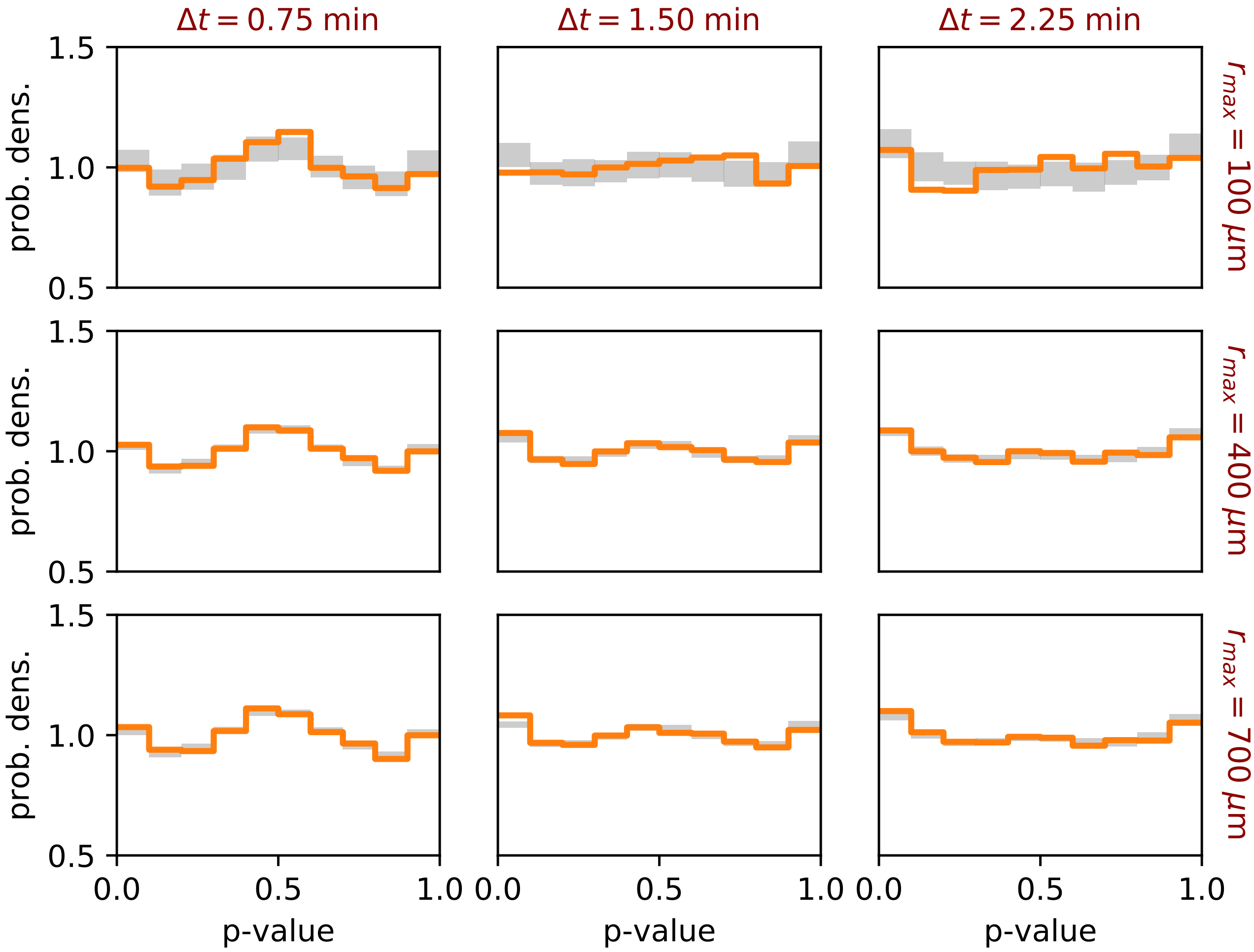

Figure 3: p-value distributions of NK cells with wild-type K562 cells (data set DS0).

p-value distributions for NK self-interaction (data sets DS0-DS8)

Our p-value method can also be applied to systems with more than two different cell types, and it is not necessary to know the 'predator-prey relations' in advance. To demonstrate this feature, we have used data sets DS0-DS8 to investigate possible interactions between the NK cells themselves. However, there is no clear evidence for such interactions (Fig. 4).

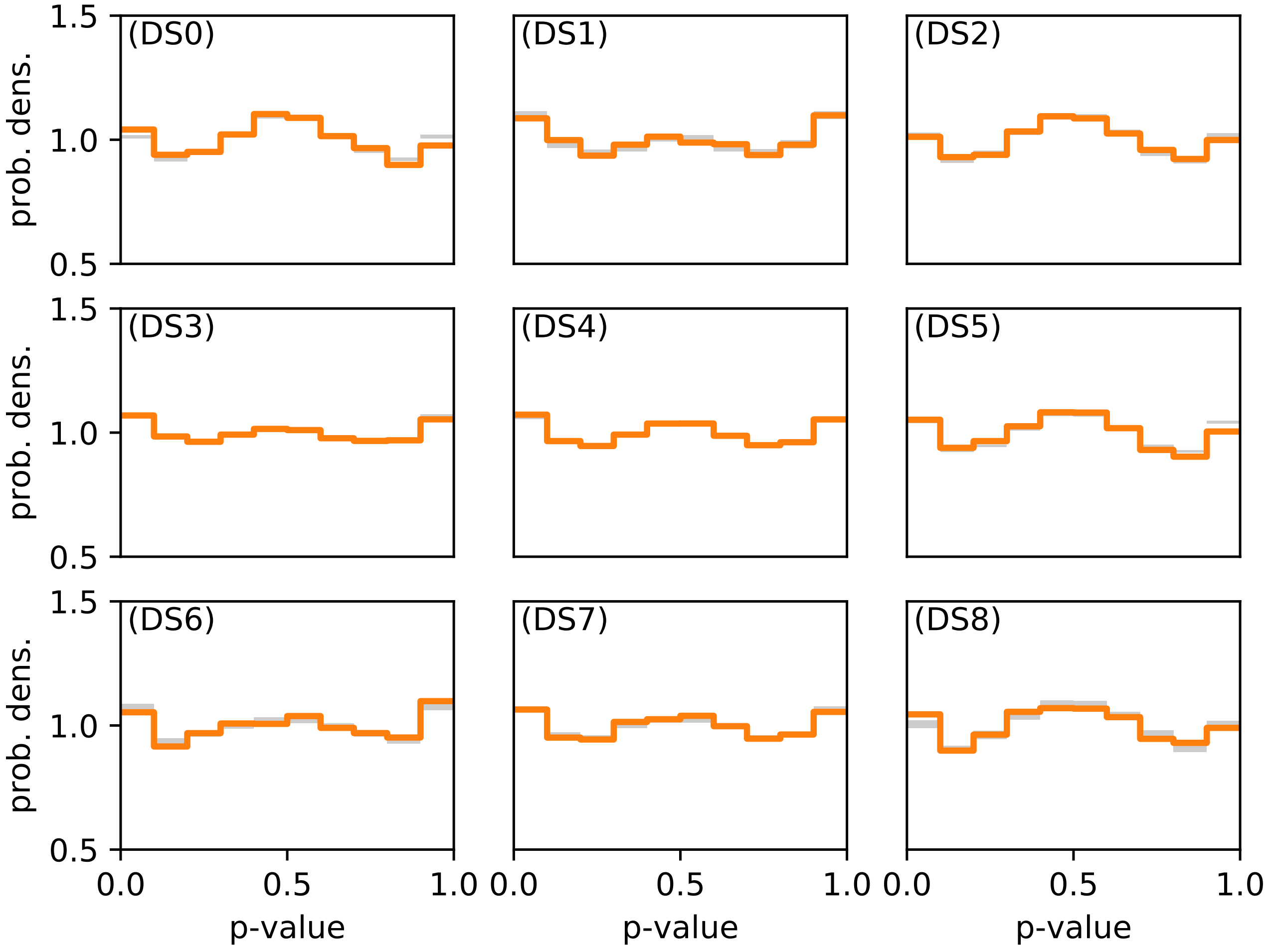

Figure 4: p-value distributions for the interactions of NK cells among themselves (data sets DS0-DS8), using a recording time interval of $\Delta t = 0.75$min and a maximum interaction radius of $r_{max} = 700\mu$m.

Video material

To compare the experimentally observed cell behavior with the models of blind search and spatial gradient sensing, we provide three videos (`https://tinyurl.com/cm-pvaluemethod`).

The video **V1.mp4** shows the tracked cells of our data set DS0. The NK immune cells are shown as red circles, the K562 tumor cells as blue circles. All cells are labeled with unique numbers. Once a tumor cell is visited by an immune cell (within a distance smaller then 30 μm), the tumor cell is considered as 'found' and is subsequently colored in gray.

The video **V2.mp4** shows a simulation that starts with the same initial configuration as in data set 0. The simulated immune cells also migrate with the same average speed and directional persistence as in the experiment (Tumor cells are assumed to by completely stationary for simplicity). This simulation assumes a target-blind random walk (blind search BLS) and produces a rate of encounters between immune and tumor cells comparable to that in data set 0.

The video **V3.mp4** shows a simulation analogous to V2.mp4, however assuming that the immune cells are chemotactically active and sense the tumor cells using spatial gradient sensing (SGS). This systematic way of approaching the targets leads to a significantly higher rate of encounters.
